# Deciphering Protein–Protein Interactions. Part I. Experimental Techniques and Databases

**DOI:** 10.1371/journal.pcbi.0030042

**Published:** 2007-03-30

**Authors:** Benjamin A Shoemaker, Anna R Panchenko

**Affiliations:** Whitehead Institute, United States of America

Proteins interact with each other in a highly specific manner, and protein interactions play a key role in many cellular processes; in particular, the distortion of protein interfaces may lead to the development of many diseases. To understand the mechanisms of protein recognition at the molecular level and to unravel the global picture of protein interactions in the cell, different experimental techniques have been developed. Some methods characterize individual protein interactions while others are advanced for screening interactions on a genome-wide scale. In this review we describe different experimental techniques of protein interaction identification together with various databases which attempt to classify the large array of experimental data. We discuss the main promises and pitfalls of different methods and present several approaches to verify and validate the diverse experimental data produced by high-throughput techniques.

## Introduction

It is now becoming clear that protein interactions determine the outcome of most cellular processes [1–4]. Therefore, identifying and characterizing protein–protein interactions and their networks is essential for understanding the mechanisms of biological processes on a molecular level. Despite the fact that protein interactions are remarkably diverse, all protein interfaces share certain common properties. Protein interactions can be classified into different types depending on their strength (permanent and transient), specificity (specific or nonspecific), the location of interacting partners within one or on two polypeptide chains, and the similarity between interacting subunits (homo- and hetero-oligomers). It has been shown that interface types are significantly different in amino acid composition so that it is possible to predict the type of interaction interface from amino acid composition alone [5]. Earlier structural analysis of interfaces showed that most interfaces consist of completely buried cores surrounded by partially accessible rims [6,7] with the overall size of about 1600 ± 400 Å^2^ (a “standard size” patch) [8]. It has been found that certain amino acids are preferred on protein interfaces and that the amino acid composition of the core differs considerably from the rim [6,7,9,10]. More recent models suggested that the protein binding site consists of a few independent highly packed regions, so called “hot spots,” which contribute significantly to the free energy of binding [11–13]. Hot spots were found to be structurally conserved [14], and the energetics of interactions at the hot spots have been analyzed in several studies [15–18].

In many cellular processes, proteins recognize specific targets and bind them in a highly regular manner. The specificity of interactions in these cases is determined by structural and physico–chemical properties of two interacting proteins. As a result, there should be a certain degree of conservation in the interaction patterns between similar proteins and domains. Indeed, it has been found that close homologs almost always interact in the same way and protein–protein interactions place certain evolutionary constraints on protein sequence and structural divergence [19–24]. Recent studies confirm that the total number of interaction types or modes is limited and rather small [25–27]. On the other hand, remotely related proteins/domains can have different interaction modes [21,26,28]; and the conservation of such protein interfaces is similar to the average conservation of rest of the protein [29–32].

In this review and its companion review in the April issue [33], we attempt to classify and systemize the array of experimental and theoretical data on the identification and prediction of protein interactions. In this review we focus on the generic experimental techniques for identifying protein interactions and the databases storing the information obtained from these experiments. In the second review, we present different methods to predict protein and domain interactions and discuss various challenges faced in this field with respect to limited prediction accuracy.

## Experimental Methods for Identifying and Characterizing Protein Interactions

**Figure 1:**
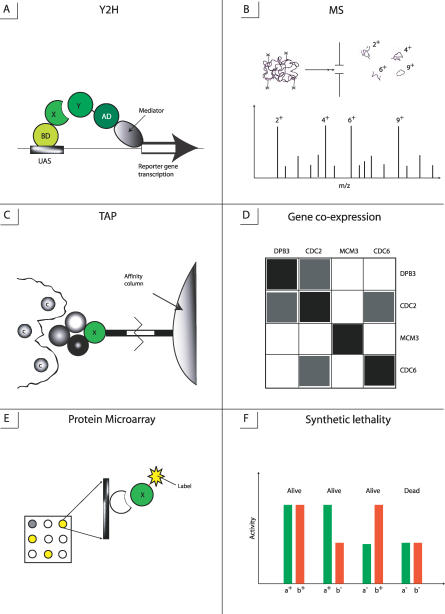
Schematic Representations of Main Experimental Techniques Used for High-Throughput Analysis of Protein Interactions (A) Y2H detects interactions between proteins X and Y, where X is linked to BD domain which binds to upstream activating sequence (UAS) of a promoter. (B) MS identifies polypeptide sequence. (C) TAP purifies protein complexes and removes the molecules of contaminants. (D) Gene coexpression analysis produces the correlation matrix where the dark areas show high correlation between expression levels of corresponding genes. (E) Protein microarrays (protein chips) can detect interactions between actual proteins rather than genes: target proteins immobilized on the solid support are probed with a fluorescently labeled protein. (F) Synthetic lethality method describes the genetic interaction when two individual, nonlethal mutations result in lethality when administered together (a^−^ b^−^).

Protein interactions can be analyzed by different genetic, biochemical, and physical methods, which are listed in Table 1 and shown in Figure 1. Some techniques enable screening of a large number of proteins in a cell, such as yeast two-hybrid (Y2H), tandem affinity purification (TAP), mass spectroscopy (MS), DNA and protein microarrays, synthetic lethality, and phage display. Other methods focus on monitoring and characterizing specific biochemical and physico–chemical properties of a protein complex.

**Table 1:**
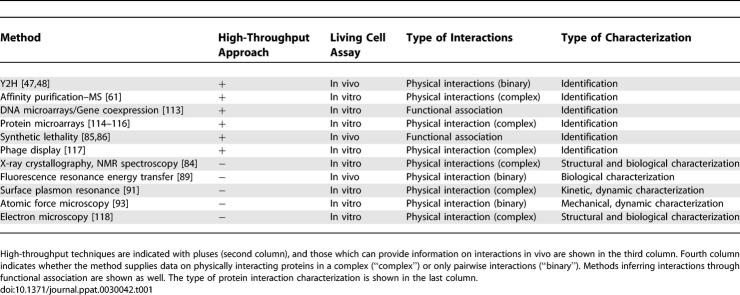
Different Experimental Methods Measuring Protein Interactions

### 

#### Yeast two-hybrid method.

The development of the Y2H technique has considerably accelerated the screening of protein interactions in vivo. Y2H is based on the fact that many eukaryotic transcription activators have at least two distinct domains, one that directs binding to a promoter DNA sequence (BD) and another that activates transcription (AD) (Figure 1A). It was demonstrated that splitting BD and AD inactivates the transcription, but the transcription can be restored if a DNA-binding domain is physically (not necessarily covalently) associated with an activating domain [34]. According to the Y2H method, a protein of interest is fused to BD (bait). This chimeric protein is cloned in an expression plasmid, which is then transfected into a yeast cell. A similar procedure creates a chimeric sequence of another protein fused to AD (prey). If two proteins physically interact, the reporter gene is activated. The most broadly used Y2H systems are GAL4/LexA-based, where the GAL4 protein controls in yeast the expression of the LacZ gene encoding beta-galactosidase. Numerous variations of Y2H have been developed including systems with several reporter genes, one-hybrid and three-hybrid systems for identifying proteins interactions with DNA and RNA [35–38], systems for detecting interactions in mammalian and prokaryotic cells, and systems for screening the interactions between membrane proteins [39–43].

For screening entire genomes, the Y2H method has been advanced into two main approaches [44–46]: matrix-based and library-based.

In the *matrix approach,* a matrix of prey clones is created where each clone expresses a particular prey protein in one well of a plate. Then each bait strain is mated with an array of prey strains and those diploids where two chimeric proteins interact are selected based on the expression of a reporter gene and the position on a plate.

In the *library approach,* each bait is screened against an undefined prey library containing random cDNA fragments or open reading frames (ORFs). Diploid positives are selected based on their ability to grow on specific substrates; and interacting proteins are determined by DNA sequencing. The first two genome-wide analyses of the yeast “interactome” revealed 692 and 841 putative interactions, respectively [47,48]. The overlap between these two experimental studies was quite small; both methods shared only 141 interactions, about 20% of the interaction data [48]. Recently, Y2H has been used to identify interactions in worm [2], fly [1], and human [49,50].

The small overlap between Y2H experiments can be explained by different factors, among them: differences in protein interaction sampling, Y2H bias towards nonspecific interactions [51], and limitations of the Y2H method itself. For example, proteins initiating transcription by themselves cannot be targeted in Y2H experiments; and the use of sequence chimeras can impose difficulties since fusion can change the structure of a target protein. In addition, protein folding and posttranslational modifications can differ between yeast and other organisms. This makes it difficult to screen proteins from mammalian and prokaryotic cells using Y2H as well as cytoplasmic and membrane proteins. To validate the quality of Y2H protein interactions in vivo*,* different in vitro techniques can be used.

#### Mass spectroscopy.

MS is a powerful method of studying macromolecular interactions in vitro. The principle of the MS method is to produce ions which can be detected based on their mass-to-charge ratios, thereby allowing the identification of polypeptide sequences [36,52,53] (Figure 1B). The problem of converting protein/peptide molecules from the condensed phase into ions in the gas phase is solved by using Electrospray Ionization (ESI) [54] and Matrix Assisted Laser Desorption Ionization (MALDI) [55,56]. Different algorithms have been developed to analyze mass spectra and to identify proteins by their sequence [57–60]. Some of them find correlations between theoretical and experimental spectra while others use de novo algorithms to infer peptide sequences from theoretical interpretation of the mass spectra. Despite the usefulness of MS for the characterization of interacting proteins, purification of protein complexes turns out to be the limiting step of their identification. To address this, TAP has been developed.

#### TAP method of complex purification.

A TAP tag consists of two IgG binding domains of *Staphylococcus* protein A and a calmodulin binding peptide separated by the tobacco etch virus protease cleavage site [61,62] (Figure 1C). A target protein open reading frame (ORF) is fused with the DNA sequences encoding the TAP tag and is expressed in yeast where it can form native complexes with other proteins. At the first step of the TAP purification, protein A binds tightly to an IgG matrix; and after washing out the contaminants, the protease cleaves the link between protein A and IgG matrix. The eluate of this first step is then incubated with calmodulin-coated beads in the presence of calcium. After washing, the target protein complex is released. The components of each complex are screened by polyacrylamide gel electrophoresis, cleaved by proteases, and the fragments are identified by MS. Comparing Y2H and TAP–MS, it should be noted that both methods generate a lot of false positives and miss a lot of known interactions. Y2H has the advantages of being an in vivo technique and of detecting transient interactions. In contrast, TAP–MS can report on higher-order interactions beyond binary and, therefore, provides direct information on protein complexes.

Several large-scale studies of protein complexes have been performed using TAP–MS and Y2H methods [2,4,63,64]. For example, Krogan et al. showed that 7,123 protein interactions identified with high confidence in yeast can be clustered into 547 protein complexes [3].

#### Gene co-expression.

Since the function of a protein complex depends on the functionality of all subunits, subunits should be present in stoichiometric amounts and gene expression levels of subunits in a complex should be related. Gene expression profiles can be provided, for example, from cell cycle experiments and expression levels of a gene under different conditions. Expression profile similarity can be calculated as a correlation coefficient between relative expression levels of two genes/proteins or the normalized difference between their absolute expression levels or calculated using other methods [65–69] (Figure 1D). The distribution of these quantities for target proteins then can be compared with the distributions for random noninteracting protein pairs. It was shown that the most obvious coexpression comes from permanent complexes such as ribosome and proteasome [65]. Several studies have tackled the problem of gene co-expression and demonstrated that interacting proteins in yeast are more likely to have their genes coexpressed compared with noninteracting proteins [65,70–77]. Moreover, it was shown that expression levels of physically interacting proteins coevolve, and coevolution of gene expression can be a better predictor of protein interactions than coevolution of amino acid sequences [78]. To infer the interactions between the genes, the DNA microarray methodology can be successfully used in the conjunction with the synthetic lethality method.

#### Synthetic lethality method.

It is not very well-understood how genetic variation influences phenotype and how genes interact with each other producing different phenotypes in different strains of the same species [77,78]. These problems can be addressed by using various genetic interaction methods, the most common of which is the synthetic lethality method (Figure 1F). The synthetic lethality method produces mutations or deletions of two separate genes which are viable alone but cause lethality when combined together in a cell under certain conditions [78–83]. Since these mutations are lethal, they cannot be isolated directly and should be synthetically constructed. Synthetic interaction can point to the possible physical interaction between two gene products, their participation in a single pathway, or a similar function. For example, synthetic lethality experiments enabled the prediction of the unknown function of the YLL049W gene as belonging to the dynein–dynactin pathway, and the bridging together of the two pathways of the parallel mitotic exit network and the Cdc14 early anaphase release pathway [83].

#### Monitoring specific protein interactions.

The most detailed information about protein interaction interfaces at the atomic level can be provided by X-ray crystallography and NMR spectroscopy, but the number of solved protein complexes remains low [84]. At the same time, the real-time characterization of interacting proteins in vivo can be achieved with various spectroscopic techniques requiring the attachment of a spectroscopic label to a target protein [87,88] (Table 1). A powerful technique in this respect is fluorescence resonance energy transfer (FRET), which can occur only if two fluorophores are located close to each other [89]. Another effective method, surface plasmon resonance (SPR), does not require spectroscopic labeling and can detect interactions between soluble ligands and immobilized receptors [90,91]; while the isothermal titration calorimetry (ITC) technique allows for direct measurement of the enthalpy of binding [92]. Recently, new methods have been developed to analyze protein interactions at the single-molecule level. For example, atomic force microscopy can fairly accurately measure interaction forces ([93]) while fluorescence techniques can characterize conformational changes in proteins upon binding [94].

#### Protein interaction networks derived from experiments.

The fast development of experimental techniques for protein interactions has enabled the construction and systematic analysis of interaction networks [1,2,95]. Interaction maps obtained for one species can be used to predict interaction networks in other species, to identify functions of unknown proteins, and to get insight into the evolution of protein interaction patterns. The interaction map analyses and comparisons are based on the observation that many interactions are conserved among species (“interologs”) [46]. Sequence-based searches for “interologs” were able to identify 16%–31% of true “interologs” (tested using Y2H system) even between remotely related species such as yeast and worm [96]. Analysis of conservation in the networks produced by gene co-expression data revealed that interologs correspond to the functionally related genes responsible for core biological processes [77]. Moreover, a multiple-species network has been constructed by identifying pairs of genes with correlated expression in different organisms. A multiple-species network has shown to perform better than a single-species network in linking together functionally related genes.

#### Verification of protein interactions.

Validation of protein interaction data is difficult; except for small datasets on protein interactions provided by the Protein Data Bank (PDB) [84] and the Munich Information Center for Protein Sequences (MIPS) [97], there is no comprehensive gold standard interaction set. Several methods have been proposed for verification of protein interaction data [66,67,76,98,99], and some of them are described here.

*Expression profile reliability method (EPR)* [66] is based on the observation that interacting proteins are coexpressed. Two distributions of expression distances are defined for noninteracting and reliably interacting proteins. The distribution of expression distances for a protein set of interest is assumed to be a linear combination of two predefined distributions with the linear coefficient that characterizes the accuracy of a given dataset.

*Paralogous verification method (PVM)* [66] is based on the observation that if two proteins interact, their paralogs most likely interact. It gives more reliability to the interaction of two families that contain a greater number of interactions between paralogous proteins. This method identified ∼40% true interactions at a 1% error rate.

*Protein localization method (PLM)* [98] defines true positives as interacting proteins that are localized in the same cellular compartment and/or interacting proteins that are annotated to have a common cellular role. PLM showed that the accuracy of experimental data strongly depends on the method with up to 50% true positives detected in Y2H experiments and up to 100% true positives detected in immunoprecipitation experiments [100].

#### Protein and domain interaction databases.

A large variety of databases exists to study binary protein interactions and the higher order interactions in protein complexes. A summary of some available databases is given in Tables 2 and 3. Different databases contain interactions obtained by direct submission from experimentalists and by mining literature and other data sources; in some cases the data is verified using automated algorithms or manual curation. In addition to direct detection of physical protein interactions, indirect methods can be used to predict the functional association between proteins or to predict the location of the interaction interface itself. There is indeed a wide range of detail characterizing the interactions available from different databases. For example, Y2H data gives the identity of interacting proteins, electron microscopy provides relative positional information of interacting proteins, and crystallography provides full atomic detail of interaction surfaces. In addition, interacting proteins can be studied either as complete units or by domains used as the units of interaction. Consequently, in this review we group all databases into protein and domain-related databases.

**Table 2:**
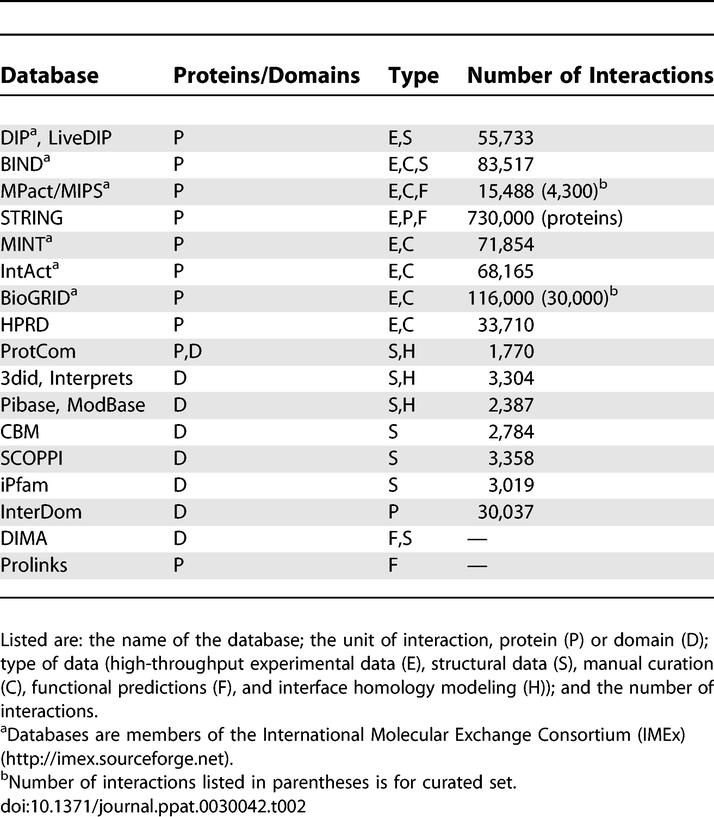
Databases Available for Searching and/or Downloading Data Related to Protein Interactions

**Table 3:**
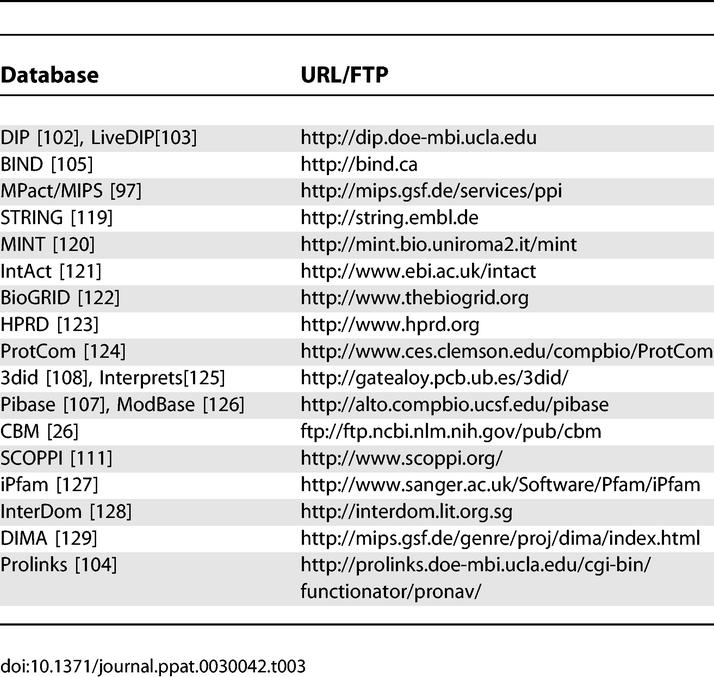
URLs and Primary Citations for Protein Interaction–Related Databases

In spite of the interaction data diversity, there exist considerable overlaps in the datasets contained in the databases, making it difficult to recommend a single resource for a particular type of information. In one effort to deal with this redundancy, the International Molecular Exchange Consortium (IMEx) has been formed in which databases agree to share their data in a consistent and timely fashion (Table 2). In addition, a standard data model has been proposed for the representation and exchange of protein interaction data [101]. A few example databases from Table 2 will now be highlighted to illustrate different types of interaction data available.

## Protein Interaction Databases

### 

#### Database of Interacting Proteins.

The Database of Interacting Proteins (DIP) contains experimentally determined protein interactions and includes a core subset of interactions that have passed a quality assessment [102]. Interaction data are obtained from the literature; PDB; and high-throughput methods such as Y2H, DNA and protein microarrays; and TAP–MS analysis of protein complexes. Several methods are employed to assess the quality of interaction data and are offered as a service for query interactions. DIP has links to a couple of related databases including LiveDIP, which records information about the state of a biological interaction, such as covalently modified, conformational, or cellular location states [103]. Another database related to DIP is Prolinks, which brings together four methods of linking proteins: phylogenetic profiles, Rosetta Stone, gene neighbors, and gene clusters[104]. The database includes a Proteome Navigator tool to browse the linkages and view accompanying data.

#### Biomolecular Interaction Network Database.

The Biomolecular Interaction Network Database (BIND) includes high-throughput experimental datasets and protein complexes from PDB [105,106]. It contains a variety of curated experimental data. A generalized data specification handles not only various types of protein interaction data, but also protein–small molecule interactions and protein–nucleic acid interactions. An interaction viewer is provided to browse the interaction space. BIND also can distinguish different functional types of interactions.

#### Munich MPact/MIPS database.

MPact is a resource to access MIPS, which contains a manually curated yeast protein interaction dataset [97] collected by curators from the literature. The resource also includes high-throughput results for yeast, but keeps this data separate. MIPS is often used as a standard of truth database for evaluating the quality of data and the accuracy of interaction prediction methods.

## Domain Interaction Databases

### 

#### PIBASE database.

PIBASE is a database of domain interactions from the protein structure data [107]. It uses SCOP and CATH domain definitions to find putative domain interactions. Several methods are employed to remove redundancy in structural data; for example, structural comparisons of interfaces are made between domains within one structure. The database combines physicochemical properties of protein binding sites and has a link to MODBASE [108], containing models of three-dimensional structures that allow use of PIBASE for modeling of putative domain interfaces.

#### 3did database.

3did allows one to explore the details of domain interactions from protein structure data (yeast interactions are also included) [109]. For each domain, an overview is given of all its interactions with other domains, showing different interaction types. In some cases, dot plots of structural comparisons between interaction interfaces show the variance of the interactions between pairs of domain families. Database entries are also supplied with the GO-based functional annotations. InterPreTS is a Web-based service associated with 3did that predicts domain interactions based on sequence homology of query proteins to a database of interacting domains (DBID) [21].

#### Conserved Binding Mode database.

The Conserved Binding Mode (CBM) database is a collection of domain interactions from the structure data where domains are defined by the Conserved Domain Database [110]. Unlike other structure-based databases, domain interactions are grouped by geometry into conserved interaction modes for each pair of domain families across all PDB structures [26]. Structural superpositions are used to infer CBMs from different members of interacting domain families docking in the same way. Such domain interactions with recurring structural themes have greater significance to be biologically relevant, unlike spurious crystal packing interactions. CBMs can also assist in analyzing protein interaction network topology by emphasizing connections made in a biological context. Finally, the CBM database can be used to categorize the specific interaction surfaces that have evolved from conserved domains and thereby allows for the homology modeling of protein interaction interfaces. A similar approach for grouping interaction patterns for SCOP domains was recently undertaken with the SCOPPI database [111].

#### Domain Interaction Map database.

Domain Interaction Map (DIMA) database is a domain interaction map derived from phylogenetic profiling Pfam domains [97]. Instead of looking at entire protein sequences, the algorithm compares the occurrences of domains across genomes and associates similar patterns of occurrences with functional associations. The method works well for domains with moderate information content that have distinct phylogenetic profiles.

In this paper we have reviewed a wide spectrum of experimental techniques for identifying and characterizing protein interactions; each technique can provide a piece in the puzzle of mechanisms of protein recognition [112]. Despite enormous efforts in this field, the overall picture is still incomplete, which is not surprising given the enormous complexity of a cell. Indeed, proteins can behave differently in different parts of the cell, and many proteins form transient complexes that are difficult to identify. Moreover, evolutionarily conserved proteins have much better coverage in experiments than the proteins restricted to a certain organism. The low coverage together with the small overlap between different experimental methods calls for the development of theoretical approaches for interaction data verification and prediction, the topic we address in our companion review [33]. 

## Abbreviations

AD: domain that activates transcription
BD: domain that directs binding to a promoter DNA sequence
BIND: Biomolecular Interaction Network Database
CBM: Conserved Binding Mode database
DIP: database of interacting proteins
MS: mass spectroscopy
TAP: tandem affinity purification
Y2H: yeast two-hybrid
